# GASTROGASTRIC FISTULA AFTER ROUX-EN-Y GASTRIC BYPASS: A CASE REPORT AND REVIEW OF LITERATURE

**DOI:** 10.1590/0102-672020190001e1509

**Published:** 2020-08-24

**Authors:** Khaled ALYAQOUT, Sulaiman ALMAZEEDI, Mohanned ALHADDAD, Evangelos EFTHIMIOU, Marcelo de Paula LOUREIRO

**Affiliations:** 1 Chelsea and Westminster Hospital Trust, General Surgery, London, United Kingdom; 2 Jaber Hospital Kuwait, General Surgery, Kuwait; 3 Positivo University, Minimally Invasive Surgery, Curitiba, PR, Brazil

**Keywords:** Fistula, Stomach, Bariatric surgery, Fístula, Estômago, Cirurgia bariátrica

## INTRODUCTION

In 1994 Cucchi et al.^7^ first published a paper identifying gastrogastric fistulas (GGF) as a complication of open divided Roux-en-Y gastric bypass (RYGB). The findings showed that GGF develop regardless of the remnant division from the pouch. Some authors attribute GGF to technical failure, early postoperative leaks or even marginal ulcers. Furthermore, diagnosis is usually difficult and requires a high index of suspicion, mainly due to a lack of pathognomonic symptoms and signs^14^. As of today, there is no consensus regarding an optimal diagnostic pathway for GGF, and management is usually patient tailored^13^ ^,^ ^14^. 

In this paper, we present a case of a lady treated at our centre with recurrent GGF, and provide an up-to-date literature review of the topic.

## CASE REPORT

Woman of 42 year-old with a BMI of 44 kg/m^2^ underwent a previous anti-gastric anti-colic RYGB using a circular staple for the gastro-jejunostomy anastomosis (GJA) in Jaber Hospital, Kuwait. Intra-operatively the anvil had an incomplete anastomotic stapler doughnut; however, both the intra-operative methylene blue and air tests were negative. The anastomotic line was the buttressed with 2-0 absorbable sutures. Two days post-operatively the patient developed acute abdominal pain, tachycardia and fever, with a water-soluble contrast study suggesting a GJA leak. A subsequent diagnostic laparoscopy however, was unremarkable, and she was managed conservatively. Seven years later, she again presented complaining of a two month history of progressive epigastric and retrosternal chest pain. Blood investigations showed mild leucocytosis and hyper-amylasemia. Gastroscopy demonstrated bile entry to the gastric pouch, with a corresponding 6-7 mm GGF. A barium swallow confirmed GGF, with no other fistulas nor strictures. She was managed endoscopically with one endo-clip applied to GGF, and its edges were burned using argon plasma coagulation.

**FIGURE 1:**
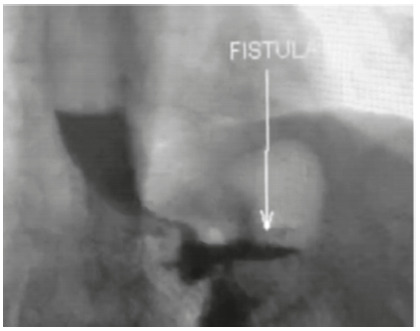
Barium swallow showing the passage of contrast to the remnant stomach

After three years she was attended again with abdominal pain and distention, associated with weight regain and vomiting. A barium swallow confirmed recurrence of the fistula (Figure 1), and gastroscopy showed a large fistulous opening measuring 15-20 mm, not feasible for endoscopic intervention.

**FIGURE 2:**
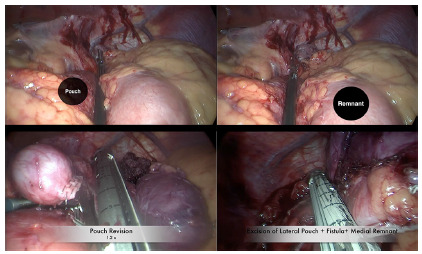
Resizing of the gastric pouch and resection of the GGF

She, therefore, underwent a laparoscopic repair whereby the GJA was first taken down, followed by excision of the lateral edge of the gastric pouch and medial edge of gastric remnant (Figure 2). The GJA was then refashioned using a hand-sewn end to side technique. She had a six day hospital stay due to surgical site infection, and afterwards was discharged.

## DISCUSSION

### Incidence

Historically one in two non-divided bypasses was complicated with GGF‎2. However, over the past decade there has been an overall decline in the incidence. Since 2010 the incidence ranged between 0-1.18%^4^ ^,^ ^15^ ^,^ ^16^ ^,^ ^18^.

The true incidence of GGF is difficult to ascertain due to the fact that some patients remain asymptomatic. Nevertheless, in one study, routine upper gastrointestinal swallow performed on all RYGB patients 48 h postoperatively found the incidence to be 1.7% among 417 consecutive patients^10^.

Table 1 summarizes the incidence of GGF in published series to date.

**TABLE1:** Overview of post-Roux-en-Y gastric bypass gastrogastric fistula incidence

Author	Year	Total divided RYGB (open+laparoscopic )	GFF	Incidence Rate (%)
Cucchi et al.	1995	100	6	6
Maclean et al.	1997	123	4	3
Corrodeguas et al.	2005	1292	15	1.2
Gumbs et al.	2006	282	5	1.8
Tucker et al.	2007	1763	27	1.5
Salimath et al.	2009	1796	20	1.1
Yao et al.	2010	366	0	0
Ribeiro-parenti et al.	2017	1900	9	0.5
Chahine et al.	2018	1273	15	1.8

Many factors play a potential role in the formation of GGF. Technical failure due to incomplete separation of the proximal stomach has been hypothesized as a main culprit. This might be secondary to false perception of complete division, or improper intra-operative visualization. Staple line or GJA leaks are also believed to play a role. Other etiologies such as marginal ulceration causing GGF have been laid out. Much debate remains, however, as to whether GGF are a cause or a result of marginal ulceration^14^.

A number of anatomical GGF classification systems have been proposed in the literature, and are mostly based on the distance between the fistula and the GJA^4^ ^,^ ^15^. Chahine et al.^4^ proposed a system whereby type one GGF are high and more than 1 cm away from the GJA, while type two are low and less than 1 cm away. Of note, this classification is based solely on intra-operative findings. In another series, a similar classification system was adapted by Ribeiro-parenti et al.^15^. This however is based on a combined radiological, endoscopic and intraoperative classification. Type one fistulas were termed proximal and were more than two cm from GJA, while type two were termed distal and were less than 2 cm from the GJA.

GGF are not easily recognized because of the lack of and/or ambiguity of presenting symptoms. For example, five out of seven GGF patients were found to be asymptomatic in one series^10^. Furthermore the symptoms typically mimic those of common RYGB complications making diagnosis quite a challenge. A review of literature with the common presenting symptoms and their relative frequency is summarized in Table 2 ^1^ ^,^ ^4^ ^,^ ^6^ ^,^ ^7^ ^,^ ^15^ ^,^ ^17^.

**TABLE 2:** Common presenting symptoms in patients with gastrogastric fistula

	Abdominal Pain	Weight regain	Nausea	Vomiting	Reflux/heartburn	Diarrhea	Bleeding	Failure to thrive	Fever
Chahine et al.	73.3%	80.0%	86.6%	N/A	40.0%	13.3%	N/A	N/A	N/A
Ribeiro-Parenti et al.	77.7%	55.5%	N/A	11.1%	N/A	N/A	11.1%	N/A	N/A
Corcelles et al.	72.2%	50.0%	N/A	50.0%	73.0%	N/A	5.5%	22.0%	N/A
Tucker et al.	37.0%	33.0%	N/A	18.5%	N/A	N/A	11.1%	N/A	N/A
Cucchi et al.	100.0%	N/A	83.0%	66.6%	N/A	33.0%	N/A	N/A	100.0%
Campos et al.	51.6%	100.0%	N/A	N/A	N/A	N/A	9.6%	N/A	N/A

Diagnostic tools for GGF are broadly divided into two categories: radiological and endoscopic. Radiologically, upper gastrointestinal series and computed tomography (CT) scans can serve as important tools in both diagnosis of GGF and delineation of anatomy^14^. 

Today, upper gastrointestinal series remains the gold-standard radiological investigations for GGF^8^. This, however, is changing. CT recognition of GGF continues to evolve, and is playing a bigger role as more CT specific findings are defined with time. For example, in a retrospective study by Gao et al.^8^ the relative attenuation ratio of contrast in the remnant stomach on CT scan was found to be 100% sensitive in GFF diagnosis.

Endoscopy has also proven itself to be an important tool in diagnosis of GGF, but the yield is dependent on the operator’s awareness of the possibility of GGF. This awareness comes in two forms, either a pre-procedural clinical or radiological suspicion, or an intra-procedural finding such as a marginal ulcer^1^ ^,^ ^4^ ^,^ ^5^.

Many surgeons today have adapted a combined endoscopic/radiological approach, and this appears to enhance diagnostic accuracy^4^ ^,^ ^6^ ^,^ ^14^ ^,^ ^15^ ^,^ ^17^. To date, however, there is a lack of strong evidence proving the superiority of one modality over another. In one series, the sensitivity of gastroscopy was slightly superior to upper gastrointestinal series in diagnosing GGF (72.2% vs. 70% respectively), but was found to have a lower sensitivity in another study (66.6 vs. 100% respectively)^6^ ^,^ ^15^. Furthermore, in a third study, gastroscopy detected less GGF than CT with oral/intravenous contrast (73.3% vs. 100%)^4^.

Although once a cornerstone in GGF management, the therapeutic role of medical treatment seems to be regressing. The mainstay of medical treatment is lifestyle modification, such as smoking cession, sucralfate in the presence of marginal ulcer and pharmacotherapy using proton pump inhibitors^14^. In one series, GGF completely resolved in 18.2% of patients using conservative treatment alone, while 45.5% were symptomatically controlled^1^. It is therefore reasonable to discuss medical options with patients, and offer it to those deemed high-risk or refusing invasive interventions.

A number of endoscopic techniques are used to seal GGF including endoscopic clips, stents, and suturing^12^ ^,^ ^14^. Most of these interventions have yet to be proven durable intermediate-term closure techniques, and literature lacks evidence on their long-term efficacy. In one study, however, Pauli et al.^14^. achieved a relatively high success rate, with half of the patients showing promising intermediate-term results using endoscopic endo-clip. The authors also concluded that GGF size was found to be inversely related to the success rate, with GGF larger than 1 cm less likely to heal endoscopically.

Endoscopic stenting has also shown success in treating GGF post-RYGB, with reported heal rates of up to 76%^12^. This, however, is challenged with a high incidence of stent migration (30.6%), and an increased risk of perforation. All in all, endoscopy has proven itself to be very useful in the management of GGF, but future advances in endoscopic technology might prove it to be even more successful in the days to come.

To date, surgery remains the most definite treatment of GGF. Surgical techniques are variable, and there is no consensus regarding optimal surgical choice^6^. In general, surgical management falls into one of three categories: simple resection of the fistula, resection of the fistula with revision of the GJA, resection of the fistula with remnant gastrectomy +/- revision of GJA^4^ ^,^ ^6^ ^,^ ^15^.

The location of fistula can play a major role in determining the extent of surgery^4^ ^,^ ^15^. Surgeon preference as well as pre and intra-operative findings may also determine the need to either revise the GJA and/or perform a gastrectomy^6^. Other operative adjuncts include an omental or jejunal interposition, theoretically reducing GGF recurrence postoperatively^2^.

All in all, although it remains a rare occurrence after RYGB it is important for the bariatric surgeon today to recognize GGF as a potential complication of the procedure. Since many patients with it do not present any typical signs, a high index of suspicion should be raised when post-RYGB patients present with symptoms such as abdominal pain and weight regain. Understanding the pathogenesis can help in potentially avoiding GGF, and knowledge of the diagnostic modalities aid in swift diagnosis and subsequent tailoring of patient-specific optimal management protocols. Further research is needed to set global guidelines and reach a consensus on treatment algorithms.
